# Pilot phase of an internet-based RCT of HIVST targeting MSM and transgender people in England and Wales: advertising strategies and acceptability of the intervention

**DOI:** 10.1186/s12879-019-4247-1

**Published:** 2019-08-08

**Authors:** T. Charles Witzel, Michelle M. Gabriel, Leanne McCabe, Peter Weatherburn, Mitzy Gafos, Andrew Speakman, Roger Pebody, Fiona M. Burns, Chris Bonell, Fiona C. Lampe, David T. Dunn, Denise Ward, Justin Harbottle, Andrew N. Phillips, Sheena McCormack, Alison J. Rodger

**Affiliations:** 1 Sigma Research, Department of Public Health, Environments & Society, London School of Hygiene and Tropical Medicine, 15-17 Tavistock Place, London, WC1H 9SH UK; 2 MRC Clinical Trials Unit at UCL, London, UK; 3 Department of Global Health and Development, London School of Hygiene and Tropical Medicine, London, UK; 4 Institute for Global Health, UCL, London, UK; 5 NAM Aidsmap, London, UK; 6 Royal Free London NHS Foundation Trust, London, UK; 7 Department of Public Health, Environments & Society, London School of Hygiene and Tropical Medicine, London, UK; 8 SH:24, London, UK

**Keywords:** HIV self-testing, Men who have sex with men, Transgender people, Randomised controlled trial, Online service delivery, Implementation science, Process evaluation

## Abstract

**Background:**

The SELPHI study (An HIV Self-Testing Public Health Intervention) is an online randomised controlled trial (RCT) of HIV self-testing (HIVST). The aim of this study was to assess the feasibility of recruiting UK men who have sex with men (cis and trans) and trans women who have sex with men to the SELPHI pilot, and the acceptability of the HIVST intervention used among those randomised to receive a kit.

**Methods:**

A mixed-methods approach to assessing trial feasibility and intervention acceptability was taken, using quantitative data from advertising sources and RCT surveys alongside qualitative data from a nested sub-study.

**Results:**

Online recruitment and intervention delivery was feasible. The recruitment strategy led to the registration of 1370 participants of whom 76% (1035) successfully enrolled and were randomised 60/40 to baseline testing vs no baseline testing. Advertising platforms performed variably. Reported HIVST kit use increased from 83% at two weeks to 96% at three months. Acceptability was very high across all quantitative measures. Participants described the instructions as easy to use, and the testing process as simple. The support structures in SELPHI were felt to be adequate. Described emotional responses to HIVST varied.

**Conclusions:**

Recruiting to a modest sized HIVST pilot RCT is feasible, and the recruitment, intervention and HIVST kit were acceptable. Research on support needs of individuals with reactive results is warranted.

**Electronic supplementary material:**

The online version of this article (10.1186/s12879-019-4247-1) contains supplementary material, which is available to authorized users.

## Background

Late diagnosis of HIV infection and on-going HIV transmission in UK men who have sex with men (MSM) and transgender people are enduring public health challenges [1, 2]. Despite recent successes in reducing HIV incidence through combination prevention initiatives including expansion of testing, many MSM continue to test sub-optimally and up to 25% have never tested [1, 3–5]. Few data exist on HIV testing among transgender people, although transgender women are disproportionately affected by HIV [6, 7] and evidence suggests innovative HIV prevention interventions are key to reducing incidence in this group [7].

HIV self-testing (HIVST) is a recent approach whereby an individual tests themselves and reads their own result using a rapid diagnostic test. There is an emerging evidence base suggesting that HIVST has the potential to improve access and overcome barriers to testing through reducing stigma and privacy concerns as well as increasing convenience and ameliorating geographical barriers in areas underserved by other HIV testing opportunities [8, 9]. It also provides flexibility in intervention design: components and delivery mechanisms can be adapted depending on the target population [8]. In 2016 the World Health Organization incorporated HIVST into its Consolidated Guidelines for HIV Testing Services with the recommendation that HIVST be provided as a supplementary option alongside existing services [10].

Evidence from UK studies conducted shortly after HIVST became commercially available in 2015 suggests that HIVST is acceptable, and that MSM have preferences for blood-based tests (due to perceptions of greater accuracy) and easy access to confirmatory testing [5, 11, 12]. However, a minority of MSM with aversion to blood reported being unwilling to use a blood-based kit [11]. Home delivery of an HIVST kit is a barrier for some with concerns around domestic privacy [11]. HIVST may be more appealing to groups who do not test in line with current guidelines, which recommend annual testing for all MSM, or more frequent testing if at increased risk [5]. Evidence also suggests that challenges relating to instructions and lack of familiarity with testing procedures may present barriers to use, particularly initially [11]. Little data exists on trans populations, although a study in San Francisco found HIVST was acceptable and feasible for trans women [13]. In addition, a small number of trans women have accessed England’s national HIV self-sampling (HIVSS) service indicating that testing outside clinics may be preferable to some [2].

HIVSS, whereby a person takes their own sample and returns it to a lab that then processes it and provides a result, is the technology perhaps most analogous to HIVST. HIVSS has suffered from sub-optimal sample returns, with testing completion rates around 55% in service evaluations in the UK [14, 15]. Evidence suggests this relates to complicated sampling procedures which are not always feasible or acceptable to the target populations, including taking a sufficiently large blood sample to facilitate testing [16, 17]. If those accessing HIVST face similar barriers in performing tests this could threaten the aspiration of increasing testing uptake and frequency through the provision of this novel technology. Further, although the expansion of commercially available HIVST has seen moderate levels of uptake in the USA [18], HIVST may fulfil different roles for populations such as in the UK where HIV testing services are very well developed. This is especially true should HIVST also be provided at no cost, as with the vast majority of existing HIV testing models in the UK.

The SELPHI study (An HIV Self-Testing Public Health Intervention) is an online randomised controlled trial (RCT) being conducted between 2017 and 2020 which aims to assess whether HIVST can; (i) increase rates of diagnosis in those with prevalent HIV infection and ii) reduce the time between infection and diagnosis for those at risk of incident infection. The primary outcomes are ascertained through linkage to the national HV surveillance systems indicating confirmatory testing and linkage to care. SELPHI aimed to recruit 10,000 MSM (cis and trans) and trans women. Participants were recruited through advertising on geo-location social-sexual networking applications and Facebook. Initial baseline randomisation was to an offer of postal delivery of an HIVST kit accompanied with a follow-up survey or to no HIVST.

Intervention conceptualisation was underpinned by the COM-B model of behaviour change [19, 20]. COM-B is a systematically developed model which consolidates 19 pre-existing frameworks, positing that alterations in capability, opportunity and motivation are key to successful behaviour change interventions [20]. This model was chosen because of its simplicity and flexibility, and because of its use in HIV prevention interventions as well as interventions which include the provision of technologically assisted behaviour change [21–26]. The pilot phase of SELPHI ran from February to May 2017 and aimed for 1,000 recruits, from the overall target of 10,000.

Evidence on HIVST intervention implementation feasibility and acceptability in high income settings to date has focused on small scale demonstration studies distributing small numbers of kits with limited follow-up [27, 28]. The SELPHI pilot provides an opportunity to generate evidence about whether large-scale implementation of an online HIVST RCT is feasible in high-income settings. Usability of HIVST, defined as “the extent to which a product can be used by specified users to achieve specified goals with effectiveness, efficiency and satisfaction in a specified context of use [29]”, can also be assessed. It is also vital to understand intervention acceptability among those who receive the intervention to inform practitioners, policy makers and commissioners. This study, which is grounded in implementation science, will be useful in a range of contexts with similar health system features and HIV epidemics.

The aim of this study is therefore to assess the feasibility of recruiting to an online HIVST RCT in which participants are randomised to receive a free kit or not, and the acceptability of the HIVST intervention used among those randomised to receive it. We consider key questions related to advertising performance, reach, uptake, kit usability and end user reception. We use a mixed methods approach examining the feasibility of recruitment, the motivations of SELPHI participants, and the usability and acceptability of the kit itself. Theoretically, this work is informed by COM-B, a behaviour change model which is often used to explore acceptability and to conceptualise intervention components and how they may work together to produce behaviour change [20, 22, 30, 31].

## Methods

This mixed-methods study follows a data integration approach termed by Moran-Ellis et al. as following a thread [32]. As such key areas of inquiry were identified from quantitative RCT data; these were then used to guide the focus of the analysis of the in-depth interviews. This has allowed us to generate additional nuance in responding to questions about feasibility and acceptability.

### RCT study procedures

The pilot was designed to test the RCT recruitment strategy and the procedures in place for the full online trial. The pilot also tested the likely acceptability of the intervention, especially the usability of the chosen HIVST kit, its delivery mechanisms and the support offered for its use. Full details of the RCT methods can be found in the published protocol [33].

Eligible participants were men (cis and trans) and trans women; reporting lifetime anal sex with a man; not known to be HIV positive; aged 16 years and older; resident in England or Wales; willing to provide name, date of birth, postal and email address; consent to linkage with surveillance and clinic databases and not previously enrolled to the study.

The recruitment strategy utilised adverts placed in geo-location social-sexual networking applications (apps) (Grindr, Growlr, Scruff & Hornet) as well as targeted Facebook advertising. Free advertisements were placed on the Facebook page of a transgender focused clinical service. Recruitment sources were chosen based on previous experience, and through consultation with voluntary sector organisations. Grindr was chosen as it has the largest market share in the UK, with Hornet targeting a similar group. Growlr caters to a largely older sub-group of MSM, while Scruff is ostensibly most used by hirsute MSM and their admirers. Some adverts targeted a national audience, while others took a city or regional approach. Messaging was devised drawing learning from earlier formative work [11, 34], and with participant and public involvement (PPI) representatives. Key themes regarding barriers and facilitators to recruitment were identified, and two members of the study team met with PPI co-chairs to develop specific advertising messages. Adverts focused on all COM-B domains: capability was addressed through promoting ease of HIVST use; opportunity was addressed through highlighting the HIVST kits were available at no cost; and motivation was enhanced through highlighting privacy and appealing to altruism to take part in a study.  Some messages specifically highlighted trans eligibility. Advertisements appeared as sponsored posts, as direct inbox messages, as pop-up messages and as banners.

Participants were directed to a registration survey requiring informed consent and confirming eligibility, and then to an enrolment survey via email. Ineligible participants (and those not randomised to HIVST) were offered additional information on HIV testing. The enrolment survey asked additional demographic and behavioural questions. Participants were randomised 60:40 to receive an HIVST kit (baseline test [BT]) or to no kit offer (no baseline test [nBT]). Kits were distributed by post, directly to the given address by the test manufacturer (BioSure™).

Two weeks after enrolment, participants randomised to receive the HIVST kit were emailed an online follow-up survey asking if the kit had been used (and if not, why not), what the test result was, and whether further care was accessed. Two reminders were sent.

Three months after randomisation a survey was emailed to all participants asking for information on testing and risk behaviour in the intervening period. Two reminders were sent. Participants randomised to BT were also asked questions about their experiences with HIVST. They ranked on a 5-point scale their agreement with statements related to acceptability and usability of the kit: 1) the instructions were easy to use; 2) performing the test was simple; and 3) my overall experience was good.

### Intervention development

The intervention being trialled was linear. The recruitment messages being tested were both part of the intervention and trial process, but in a scaled-up intervention delivering HIVST these would be adapted accordingly. A brief HIV risk assessment was conducted through behavioural questions in the enrolment survey. The kit and accompanying sleeve were then delivered and two weeks later a follow-up survey asked about kit use and the test result. Those who reported not receiving a kit had a new delivery arranged. These components (advertisement, risk assessment, kit and two-week follow-up) were defined as the intervention as all were theorised to increase engagement with HIV testing through COM-B channels.

**Fig. 1:**
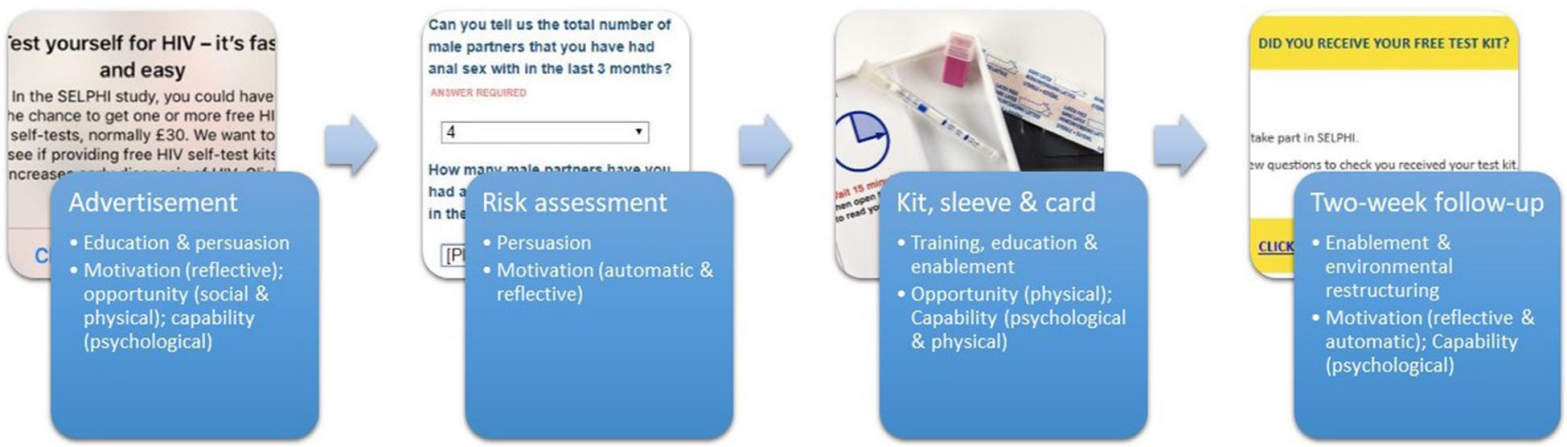
Intervention diagram with COM-B categorisation and targeted domains

Formative work was central to intervention development. Focus groups with MSM and key informant interviews identified specific barriers to uptake and use of the HIVST which the SELPHI intervention development was attentive to ameliorating, using COM-B. These efforts were also used in developing appropriate messaging for advertising. Figure 1 provides a visualisation of intervention components with a description of the intervention functions and the COM-B domains they seek to affect.

**Fig. 2:**
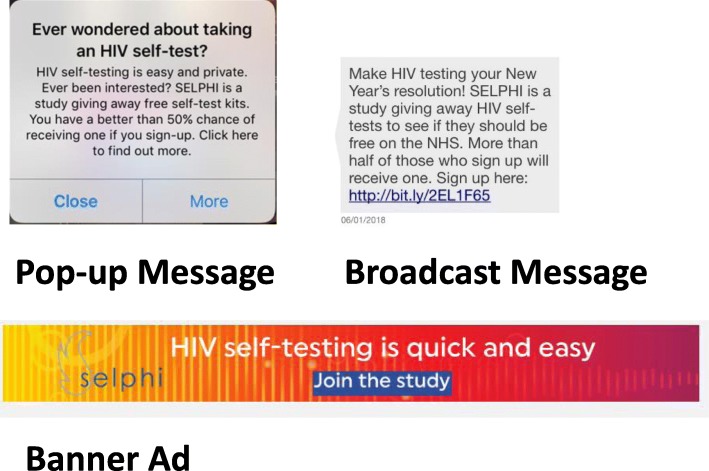
Advertising samples

Anticipated concerns regarding ease of use, coded in COM-B as capability (physical), were addressed in advertisements (see Fig. 2 for examples). This combined intervention approaches described as persuasion and education in COM-B [20], enhancing motivation by minimising concerns regarding ease of use and highlighting privacy and convenience. Issues concerning lack of knowledge in using HIVST were also identified. The COM-B model codes this as psychological capability; the behaviour change wheel then suggests intervention functions such as education, training and enablement might be useful [20]. This was alongside an observed preference for additional supportive information beyond what was provide in the original BioSure™ kit. This necessitated the development of a sleeve over the box holding the kit to provide support information (education), as well as behavioural support (enablement) which was highlighted in the two-week follow-up survey. The sleeve also provided signposting to a free telephone helpline and website for HIV and sexual health information (enablement).

In order to increase engagement with HIV testing generally, a risk assessment was included in the enrolment survey to provide a reflective experience examining personal risk. It was theorised that this approach (persuasion) can increase motivation (reflective and automatic) [20].

Formative research also identified issues with the instructions and packaging of an earlier iteration of the kit, both of which reduced motivation to access HIVST and capability when doing so. The kit instructions were reformatted by the manufacturer before implementation began, effectively addressing this issue. This intervention component is theorised as training (the imparting of skills) in the COM-B system [20].

The broader HIVST literature identifies support issues as a key concern in HIVST delivery, a concern also identified in our formative work [8, 11, 35]. This informed the provision of enhanced support information via our kit sleeve produced in collaboration with our community advisory group co-chairs (see Additional file 1). The two-week follow-up survey was also designed to counter this concern. If a participant reported a positive result here, they were directed to a page providing information on how to find their local HIV clinic. This same page was linked to from the three-month survey. Additional information about receiving a positive result was provided on the SELPHI website.

### Data handling, generation & analysis

Data pertaining to advertising reach was recorded for all adverts, then pooled according to platform. The click conversion rate (proportion of those clicking on the advert who subsequently registered) was calculated. Eligible and ineligible registrations as well as the number of successful randomisations were tabulated. Registration conversion was calculated by deriving the proportion of eligible registrations who filled in the enrolment survey and were subsequently randomised.

Baseline demographic and behavioural profiles were tabulated overall and by recruitment source. Variables considered were age (both continuous and 10 year bands), gender, sexual orientation, ethnicity (recoded from standard UK ethnicity codes into white, Asian, black & other), highest educational qualification (low: GCSEs and below; medium: A-levels or equivalent, higher education below degree level; high: degree or higher), HIV testing history (tested in preceding 12 months; tested more than 12 months ago; never tested), and condomless anal intercourse (CAI) in the preceding 3 months. Participant demographic and behavioural characteristics were compared between recruitment sources using chi-squared tests or a Kruskal-Wallis test for age.

Responses to the 2-week survey were summarised by proportion who completed the survey, proportion who received the kit, and the proportion who subsequently used the kit.

Kit use was summarised again from the 3-month survey alongside acceptability variables pertaining to instructions, simplicity of test performance and overall experience.

### Qualitative data

A qualitative study was undertaken with 10 cis-gender MSM participants during the pilot in order to examine intervention acceptability in greater depth. Participants were sampled purposively from those randomised to receive an HIVST kit. Sampling aimed to be diverse with regard to testing history: whether an individual had tested in the 12 months before joining SELPHI; not tested in the preceding 12 months; or never previously tested for HIV. Efforts were made to ensure sample diversity with regard to demographic features, especially education.

A semi-structured interview topic guide was developed to explore questions from formative research [11, 34], including issues related to capability, HIVST potential, anticipated responses and acceptability and mapped onto COM-B. The guide covered HIV testing history, motivations for joining and experiences of the SELPHI RCT, questions related to using HIVST and emotional responses.

Interviews were conducted over the phone or through Skype, and participants were electronically given a £30 incentive. All interviews were audio recorded and transcribed.

A thematic framework was developed for analysis, fusing the approaches described by Braun and Clarke [36] and Richie and Spencer [37]. This inductive process involved familiarisation with the transcripts and drawing out emerging themes. These themes were arranged into groups, with higher-level themes emerging from sub-themes, both organised hierarchically, and again mapped onto COM-B to better elucidate how acceptability of intervention components related to the behaviour change domains. The framework was piloted on two transcripts, refined, and applied to all remaining transcripts. We draw data from across this framework and report themes by COM-B domain for simplicity of interpretation.

Ethical approval for the RCT and qualitative sub-study were provided by MRCCTU and LSHTM (refs: 11945 & 9233/001). SELPHI is registered with the ISRCTN (ref: ISRCTN20312003). All RCT participants provided online written consent. Qualitative sub-study participants provided verbal recorded consent at the time of interview.

## Results

The recruitment strategy led to the registration of 1370 eligible participants through 13 advertisements across 5 platforms, of whom 76% (1035) subsequently enrolled and reached baseline randomisation. In this pilot, 631 participants were randomised to receive an HIVST kit (BT), while 404 were randomised to not receive a kit (nBT). Of those randomised to BT, 66% (415) completed the two-week follow-up survey and 64% (405) completed the first three-month survey. Overall 78% (494/631) completed at least one of these two surveys (2-weeks or 3-months).

### Recruitment strategy performance

Click conversion was highly variable, from 8% in Grindr adverts to 20% in Facebook advertising. Registration conversion ranged from 71 to 80% (mean = 76%). Cost per randomised participant varied: Hornet was cheapest (£1.66) and Grindr most expensive (£7.16). Costs were stable through this phase, with no evidence in the pilot of diminishing returns. See Table 1 for full details.

**Table 1:** Advertising source data

Recruitment source	Facebook	Growlr	Hornet	Grindr	Free/organic	Total
Number of campaigns	3	1	2	5	2	13
Advert clicks	1210	1193	Not available	6666	Not available	Not available
Registered & eligible	216	120	590	406	38	1370
Registered & ineligible	32	19	109	144	9	313
Click conversion^1^	20%	12%	Not available	8%	Not available	Not available
Randomised	173	96	433	308	27	1035
Registration conversion^2^	80%	80%	73%	76%	71%	76%
Spend per randomisation	£3.70	£2.59	£1.66	£7.16	£0.00	£3.68

^1^Click conversion: proportion of clicks leading to a registration

^2^Registration conversion: proportion of eligible registrations leading to a randomisation

### Demographic features of sample including by recruitment source

**Fig. 3:**
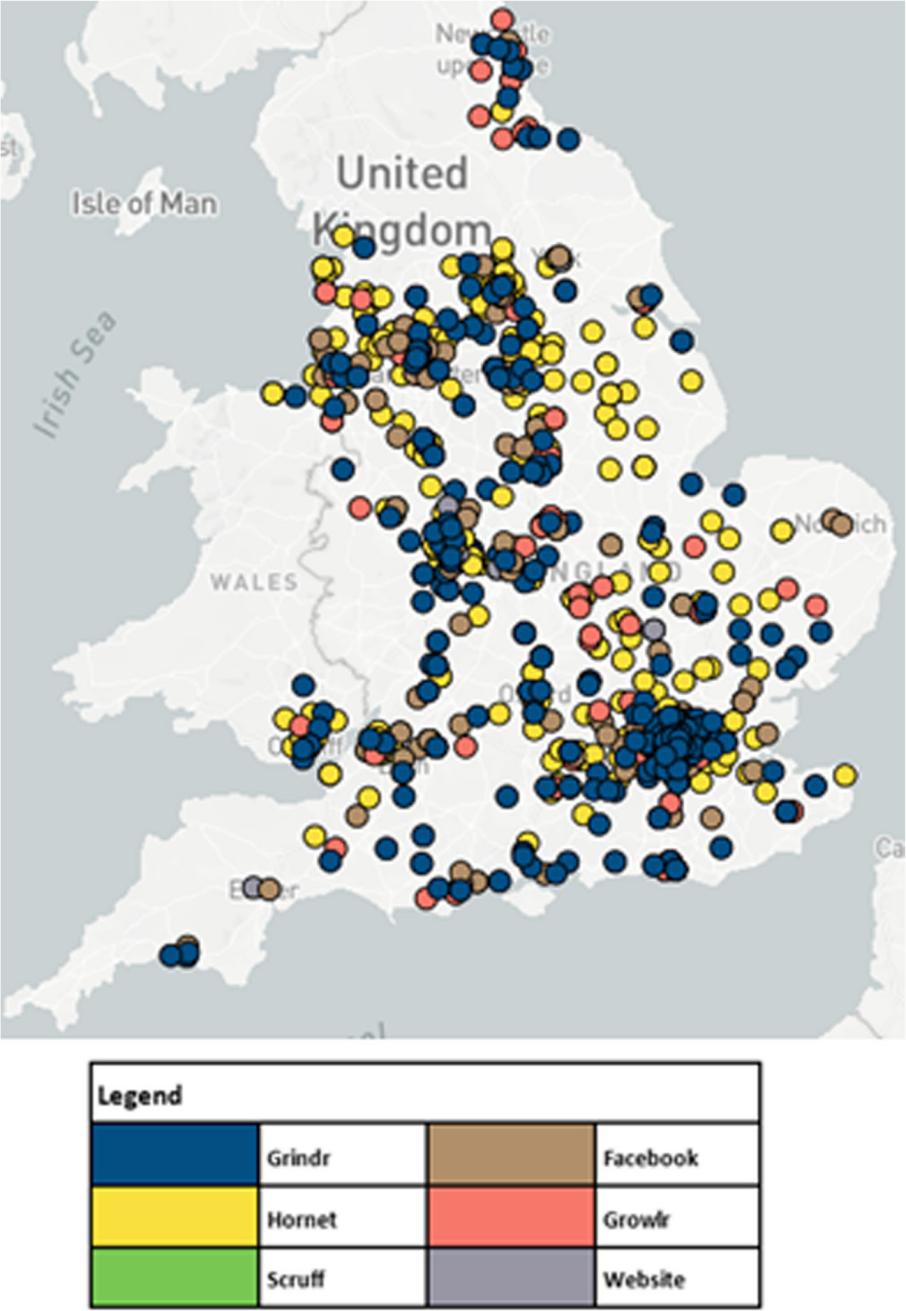
Map of randomised participants by recruitment source

Table 2 presents baseline demographics overall and by recruitment source. Figure 3 presents the geographic distribution of randomised participants with each dot representing a group of multiple randomised participant from a source, coded by colour. The recruitment strategy engaged a range of MSM, but less so trans women. Median age was 32.1 years (IQR 25.9, 41.6). Cis-gender MSM comprised the majority (99%) of the sample, as did participants of white ethnicity (89%, *n* = 921), and MSM who identified as gay (89%, *n* = 757). Most (60%, *n* = 611) were highly educated and reported CAI within the preceding 3 months (70% *n* = 726). Sixty-four percent of participants (*n* = 652) had tested for HIV in the preceding year and 14% (*n* = 141) had never previously tested. Of never tested participants, 82 (58%) reported one or more CAI partners in preceding 3 months. Table 2 presents full details of baseline demographics.

**Table 2:** Participant demographics by advert source and overall

Recruitment sources	Facebook	Growlr	Hornet	Grindr	Free/organic	Total	*p*-values
Number of campaigns		1	2	5			
N	173	96	431	308	27	1035	
Median (IQR) age (years)	29.2 (24.6, 32.8)	44.8 (34.0, 51.2)	30.8 (24.7, 39.6)	35.0 (27.6, 45.4)	33.6 (29.2, 40.0)	32.1 (25.9, 41.6)	p < 0.001
16–25 years	57 (33%)	7 (7%)	130 (30%)	60 (19%)	5 (19%)	259 (25%)	
26–35 years	112 (65%)	23 (24%)	149 (35%)	102 (33%)	10 (37%)	396 (38%)	
36–45 years	2 (1%)	22 (23%)	93 (22%)	72 (23%)	6 (22%)	195 (19%)	
46 years or older	2 (1%)	44 (46%)	59 (14%)	74 (24%)	6 (22%)	185 (18%)	
Gender identity							*P* = 0.001
Cis man	171 (99%)	96 (100%)	430 (99%)	305 (99%)	23 (85%)	1025 (99%)	
Trans man	1 (1%)	0	1 (1%)	2 (1%)	3 (11%)	8 (1%)	
Trans woman	0	0	0	1 (< 1%)	1 (4%)	2 (< 1%)	
Sexual Identity	*N* = 153	N = 72	*N* = 361	*N* = 240	*N* = 25	*N* = 851	*p* = 0.23
Gay	143 (93%)	65 (90%)	322 (89%)	204 (85%)	23 (92%)	757 (89%)	
Bisexual	8 (5%)	5 (7%)	35 (10%)	30 (13%)	1 (4%)	79 (9%)	
Other	2 (1%)	2 (3%)	4 (1%)	6 (3%)	1 (4%)	15 (2%)	
Ethnicity							*p* = 0.52
White	153 (88%)	86 (90%)	394 (91%)	263 (85%)	25 (93%)	921 (89%)	
Asian	5 (3%)	2 (2%)	13 (3%)	10 (3%)	1 (4%)	31 (3%)	
Black	3 (2%)	3 (3%)	8 (2%)	12 (4%)	0	26 (3%)	
Other	12 (7%)	5 (5%)	16 (4%)	23 (7%)	1 (4%)	23 (7%)	
HEQ	*N* = 172	*N* = 95	*N* = 425	*N* = 307	*N* = 27	*N* = 1026	*P* = 0.31
High	114 (66%)	59 (62%)	244 (57%)	174 (57%)	20 (74%)	611 (60%)	
Medium	33 (19%)	16 (17%)	94 (22%)	65 (21%)	3 (11%)	211 (21%)	
Low	25 (15%)	20 (21%)	87 (20%)	68 (22%)	4 (14%)	204 (20%)	
HIV testing history	*N* = 173	*N* = 96	*N* = 426	*N* = 304	N = 27	N = 1026	*p* = 0.09
Last 12 months	120 (69%)	54 (56%)	280 (66%)	185 (61%)	13 (48%)	652 (64%)	
12 months+	39 (23%)	27 (28%)	85 (20%)	72 (24%)	10 (37%)	233 (23%)	
Never	14 (8%)	15 (16%)	61 (14%)	47 (15%)	4 (15%)	141 (14%)	
CAI last 3 months	124 (71%)	67 (70%)	308 (71%)	206 (67%)	21 (78%)	726 (70%)	*P* = 0.58

*IQR* interquartile range, *CAI* condomless anal intercourse, *HEQ* highest educational qualification

There were significant differences in age (*p* < 0.001) and gender (*p* = 0.01) across recruitment sources, with all other variables being similar. As anticipated, Growlr recruited older participants whereas Facebook recruited younger ones. Free advertising targeted towards trans people was most effective for reaching trans participants, although numbers were small. See Table 2.

### Motivations of participants (qualitative sub-study)

Table 3 presents demographic characteristics of the qualitative sub-study. All participants were cis-MSM. When discussing their motivations for joining SELPHI, participants described three predominant motivations: i) to access HIV testing; ii) desire to use a novel technology; and iii) altruism.

**Table 3:** Qualitative sub-study sample

Demographic characteristics		Sample
Age	18–25 years	3
	26–40 years	6
	41+	1
Ethnicity	White	8
	Black	0
	Asian	1
	Other / Mixed	1
Sexual orientation	Gay	8
	Bisexual	1
	Other / undisclosed	1
Recency of HIV testing	< 12 months	2
	+ 12 months	6
	Never tested	2
HEQ	Low	3
	Medium	2
	High	5
Condomless anal intercourse preceding 3-months	0	1
	1	6
	2	2
	3–10	1

#### Accessing testing

HIVST reduced specific HIV testing barriers, thereby facilitating uptake. This was especially true for those who had never tested, and those who had not tested within the preceding twelve months. Opportunity barriers (e.g. convenience and ease of access) and motivational barriers (e.g. confidentiality and stigma) were ameliorated by HIVST.

*Sometimes people ask you what you’re coming in for,* [ … ] * if you say, ‘oh I’m coming for an HIV* [test]*’ they think you’re gay, or they think you’re disgusting, you don’t use protection, or blah, blah, blah. But it’s mainly about being labelled as something you’re not.* (23-year old bisexual man, never tested).

#### Desire to use a novel technology

Just under half of those interviewed reported being motivated to join SELPHI out of a desire to experience a novel technology or because they felt SELPHI was a new kind of study. HIVST was understood to be an evolution of HIV testing methods which was appealing to some:

*It’s an interesting one because it’s obviously very new. So you kind of think, well it’s really great.* [ … ] *You just think it’s something that’s interesting to try because it’s new technology.* (20-year old gay man, tested in last 2 years).

#### Altruism

Altruistic motivations were reported by just over half of participants with a range of testing histories. These were predominantly secondary motivations, helping support the decision to join a trial. Motivations were related to notions of good citizenship, desire to contribute to the gay community and to science more broadly.

*I find it quite interesting actually that those kinds of services are targeted towards people through Facebook because you’re kind of transpiring an audience of people who might benefit from that service. And I thought*, “Actually that’s quite smart” *because I’m in that audience. And so, I just thought*, “Yeah, I will give it a try” (34-year old gay man, tested in last 3 months).

### Kit use at two weeks and three months

Of 631 who were randomised to BT, 66% (415) completed a two-week follow-up survey. At this point, 95% (394) reported having received their kit and 83% (328) of those had used it themselves. Reasons for not using the kit were mainly that participants were planning to use it in the future (97% *n* = 64) or that participants had tested elsewhere instead (3%).

At the three-month survey, completed by 64% of eligible participants, 97% (390/403) reported having received, of which 96% (375/390) had used the kit. This indicates that although a significant minority delayed kit use, most did use the test kit by three months.

When results from both surveys were pooled, providing data for 78% of participants, 97% (477/494) received the kit and 90% (445/494) had used it. Assuming that all those participants that did not complete either of these surveys received the kit (137/ 631), but none used it, then the lowest possible estimate of kit use was 71% (445/631).

### HIVST usability and acceptability

**Fig. 4:**
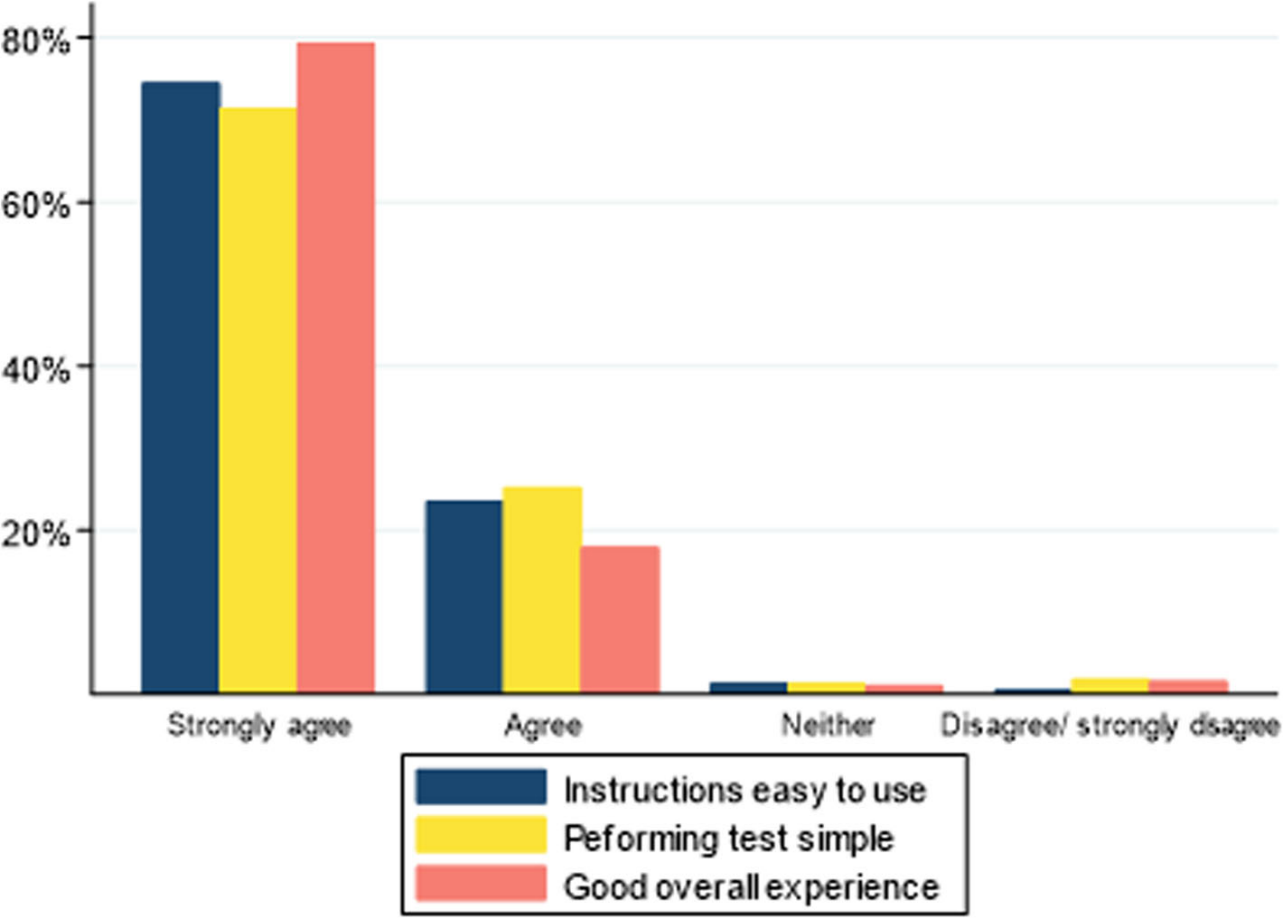
Intervention acceptability

At three months, participants reported very high HIVST usability and acceptability. Of 375 who used the kit and completed the three-month survey, 98% (362/369) found the instructions easy to understand, 97% (356/368) found the test kit simple to use and 97% (359/369) reported a good overall experience (Fig. 4).

All qualitative interview participants had used their kits to test themselves. Below we describe intervention acceptability as it relates to the main domains of COM-B: capability, opportunity and motivation.

#### Capability (physical & psychological)

Capability was the most pronounced of the three COM-B domains in our acceptability analysis, especially around test kit usability. Themes around physical capability tended to concern the instructions and using the lancet to take a blood sample. The inclusion of the two-week follow-up processes was a valued intervention element addressing psychological capability.

The instructions were generally felt to be easy to understand and interpret, although one participant felt they did not cater to a sufficiently diverse range of skills. The testing process was described as simple and the result was easy to interpret:

*Very clear and it was quite obvious as well what goes where and how to do it. It was clear. The descriptions and the pictures were easy to follow* (29 year-old man, undisclosed sexual orientation, tested in last 5 years).

Blood collection via the included lancet was a barrier for some. Those who had no previous experience of drawing blood with a lancet reported concerns about their capability to collect their own sample, although all felt with experience this would no longer be an issue.

*I actually don’t like getting my finger pricked* [ … ] *so I was most worried about the finger prick*, [ … ] *so for me that was the most difficult thing, and then I wasn’t sure if I was getting enough blood* [ … ] *but once I pricked the finger and I collected the blood then it was pretty straightforward* (31-year old gay man, tested last 4 weeks).

Capability (psychological) also emerged when discussing the support components of the intervention. Participants generally felt that the supporting information provided was adequate and did not diminish acceptability of the intervention.

*What I think relieved me of most of the anxiety was actually the kit included a card saying, at the end, if you are diagnosed with HIV then not to worry, here's what you can do. A, B, C. And if you're not, great. A, B, C. And I think the steps on that card saying, if you are, step one, step two, step three, it helped to relieve some of the uncertainty of what might happen if the test came out positive.* (16-year old gay man, not previously tested)

#### Opportunity (physical & social)

Themes related to acceptability of the entire intervention package were primarily related to opportunity (physical and social). For participants located in areas underserved by HIV testing opportunities, the kit ameliorated geographic barriers. Individuals who faced psychosocial barriers to testing felt HIVST gave them increased privacy around testing, enhancing the acceptability of the intervention.

*I’m quite a private person. I like to keep certain aspects of my life to myself and sometimes people might be bothering you to talk about things where you think,* “Well, I’m not there yet.” [ … ] *Whereas I can let that sink in and think,* “*Right, okay, now I’m ready to go and do whatever I need to do or talk to whoever I need to talk to*.” (34 year-old gay man, tested more than 12 months ago).

#### Motivation (reflective & automatic)

The dislocation of HIVST from care pathways affected acceptability through motivational channels. Despite high acceptability related to the follow-up provided, HIVST as a concept was perceived to be associated with increased anxiety relative to other testing opportunities. This was largely due to concerns about conducting a test alone, and the potential separation of initial “diagnosis” from established care pathways.

[ … ] *I think slightly the kit at home* [makes me more anxious]. *It’s almost because it’s literally taken out of your hands when you go to an STI clinic. So you don’t have to think about it as much. It’s something done to you.* (29-year-old gay man, tested within last 5 years)

One participant delayed using the kit due to anxiety and instead visited a GUM clinic, saving his HIVST for use at a later date.

The 15 min interval between conducting the test and reading the result was described as an exceptionally anxious time, especially for those who had tested due to risk.

*I was feeling nervous actually because I was thinking what about if it does come back positive. That was quite like a bit of a head scratcher, waiting for the 15 minutes, and then when 15 minutes were up and I looked at the result and I was like, oh, you know it was negative so I thought right, but then I thought well yeah, maybe I shouldn’t have worried that much, but you can’t help it* (23-year old bisexual man, not previously tested).

All participants described significant relief when reading a negative (non-reactive) result. A minority, who had more experience of HIV testing, felt that HIVST was associated with less anxiety than testing methods relying on laboratory-run tests due to the relative immediacy of results.

## Discussion

Through this mixed methods study we assessed the feasibility of recruiting MSM and trans people to the pilot phase of the online SELPHI RCT and the acceptability of HIVST, focusing mainly on the acceptability of the intervention and the usability of the kit.

Advertising performance varied according to platform by click and registration conversions and, crucially, cost. The pilot sample was predominantly white, well-educated, gay identified cis-MSM who reported CAI in the 3-months preceding and who had tested for HIV in the preceding 12-months. The pilot struggled to recruit significant numbers of trans people, particularly trans women. Our recruitment did however, reach a range of participants across demographic groups. Platforms recruited participants of a similar demographic and behavioural profile except when considering age and gender identity.

Sixty-six percent of participants completed the two-week follow-up, and 64% the three-month survey. Overall 78% of participants randomised to receive HIVST completed at least one of the two. Kit use was high, increasing from 86% at two-weeks to 96% at three months. The lowest possible estimate of kit use was 71%, assuming that all those not completing the follow-up surveys did not use their kits, which is unlikely. The kit was considered usable and the intervention was acceptable across the three dimensions interrogated (ease of use of instructions, test simple to perform and overall experience). Qualitative data provides nuance, with some participants reporting difficulty using the lancet. The relationship between HIVST and anxiety was ambiguous; individuals thought it could increase or ameliorate anxiety depending on previous HIV testing experience.

Our recruitment strategy was successful in reaching a group at risk of HIV who had not HIV tested previously, with 58% of never tested MSM reporting CAI in the 3-months preceding their enrolment. This is a key group with clear HIV prevention needs who should be a primary target for new testing interventions. The sample recruited in the pilot was comparable to previous convenience samples of MSM, as well as the ethnic make-up of the UK [38, 39]. This indicates that this type of recruitment strategy is capable of reaching a group broadly representative of UK MSM in terms of ethnicity. A group which is underrepresented when compared to national statistics is MSM of Asian ethnicity [39]. This could be due to specific privacy barriers to a postal delivery HIVST service experienced by this group, outlined in formative work [11]. Further, our sample reported similar levels of never testing (14%) to other convenience samples of UK MSM (other recent samples range between 8 and 25%), although more participants in the SELPHI pilot had tested in the preceding 12 months [38].

When compared to HIVSS return rates in the UK, participants in the pilot made use of their HIVST kits more frequently, lessening missed opportunities for testing. While with HIVSS only 55% of samples are returned for processing [14, 15], 95% reporting kit use at three months and at least 71% of kits were used overall. At this modest scale, HIVST appears to outperform HIVSS.

Acceptability and ease of use was very high, and indeed higher than in many other studies with MSM [8]. This is not without precedent, with similar levels of acceptability and reported ease of use observed in other settings [13, 40, 41]. These studies however provided oral fluid HIVSTs, which may have benefits in terms of simplicity (though have lower sensitivity and specificity) over kits which require self-collection of a whole blood sample [8, 42].

Qualitative accounts of acceptability focused on COM-B domains related to capability more than opportunity or motivation [20]. This could signify that when engaging with this novel testing technology, individuals are often doing so with questions about their own skills and capacity. These concerns may decrease with increased experience with HIVST. Indeed, using the lancet was described as difficult for many, although they managed to use it successfully despite this, and all expected this would improve with experience. An additional focus of enquiry for future study is the experience of those who have reactive tests (both confirmed as positive and subsequently confirmed negative) to better understand their experiences, support needs and any potential harms arising.

A number of changes to the trial design were made as a result of this pilot. The attrition between registration and enrolment surveys (24% of participants overall) posed significant recruitment challenges. For the main roll-out of the RCT the language in the email linking the two surveys was made more motivational, specifically highlighting altruism. In addition, all messages used in the roll-out were designed to more clearly emphasise trans eligibility. In response to the increased costs generated by attrition and to take advantage of advertising efficiencies at larger scales, national rather than regional advertising campaigns were prioritised to increase recruitment volumes. Advertisement messages were also altered, with increasing use of motivational elements. In efforts to increase survey completion rates, the number of reminders was increased from 2 to 3, and delivery times were staggered at different times of the day to account for a variety of employment patterns. These changes were supported by a PPI engagement exercise with SELPHI participants.

### Strengths and limitations

This is the first study in Europe to assess the feasibility of recruiting to an online HIVST RCT. A strength of this pilot is that the design perfectly mimics the full trial. Nevertheless, some limitations are noted. Recruited costs per participant should be treated with some caution. For one, the possibility of being randomised to the no baseline HIVST/SoC arm likely made costs per participant significantly higher than delivering free HIVST to all. In addition, recruitment costs in this pilot phase did not show evidence of diminishing returns per participant randomised, meaning that overall cost per participant could become much higher when recruiting larger numbers.

Test kit usability and intervention acceptability were extremely high when compared to other recent studies [8]. A possible explanation is the informed consent procedures in place provided a great deal of information about what a participant could expect from the study and the kit itself in a level of detail that a service might not include.

This pilot struggled to recruit large numbers of trans people compared with cis gender MSM. In addition, sexual practice among MSM is diverse. As only MSM who report lifetime anal sex were eligible for inclusion our sample may not be fully representative of the diversity in MSM sexual behaviour, as between 8 and 19% have never had anal sex [43–45]. This issue may be an especially pronounced for trans MSM and may have contributed to the low numbers of trans people recruited in this pilot phase.

Finally, while the qualitative data is illuminative, interview were conducted with a small group of participants. The data presented here should be understood as highlighting the diversity of facets of kit usability and intervention acceptability.

## Conclusion

Recruiting to this online HIVST pilot RCT was feasible, the intervention was acceptable to participants, and the kit distributed had high reported usability. Kit use was high, outperforming previous HIVSS projects in the UK. This pilot led to a number of changes to the implementation of the RCT, including national advertising and enhancing efforts to boost trial retention. Further research investigating the experiences of trans people is necessary in order to optimise future intervention approaches for this group.

## Additional files

Additional file 1: Kit sleeve design. (JPG 519 kb)

## Data Availability

RCT data will be locked and archived at the MRC Clinical Trials Unit. Requests for controlled access should be sent to mrcctu.selphi@ucl.ac.uk. Qualitative data is not available due to its sensitive and potentially personally identifiable nature.

## Abbreviations

Apps: Geospatial social/sexual networking application
CAI: Condomless anal intercourse
HIVSS: HIV self-sampling
HIVST: HIV self-testing
MSM: Men who have sex with men
PPI: Participant and public involvement
RCT: Randomised controlled trial
SoC: Standard of care
