# Supplementary figures and images for: Pilot phase of an internet-based RCT of HIVST targeting MSM and transgender people in England and Wales: advertising strategies and acceptability of the intervention
Source: BMC Infect Dis. 2019 Aug 8;19:699. doi: 10.1186/s12879-019-4247-1 (PMC6686516; doi:10.1186/s12879-019-4247-1)

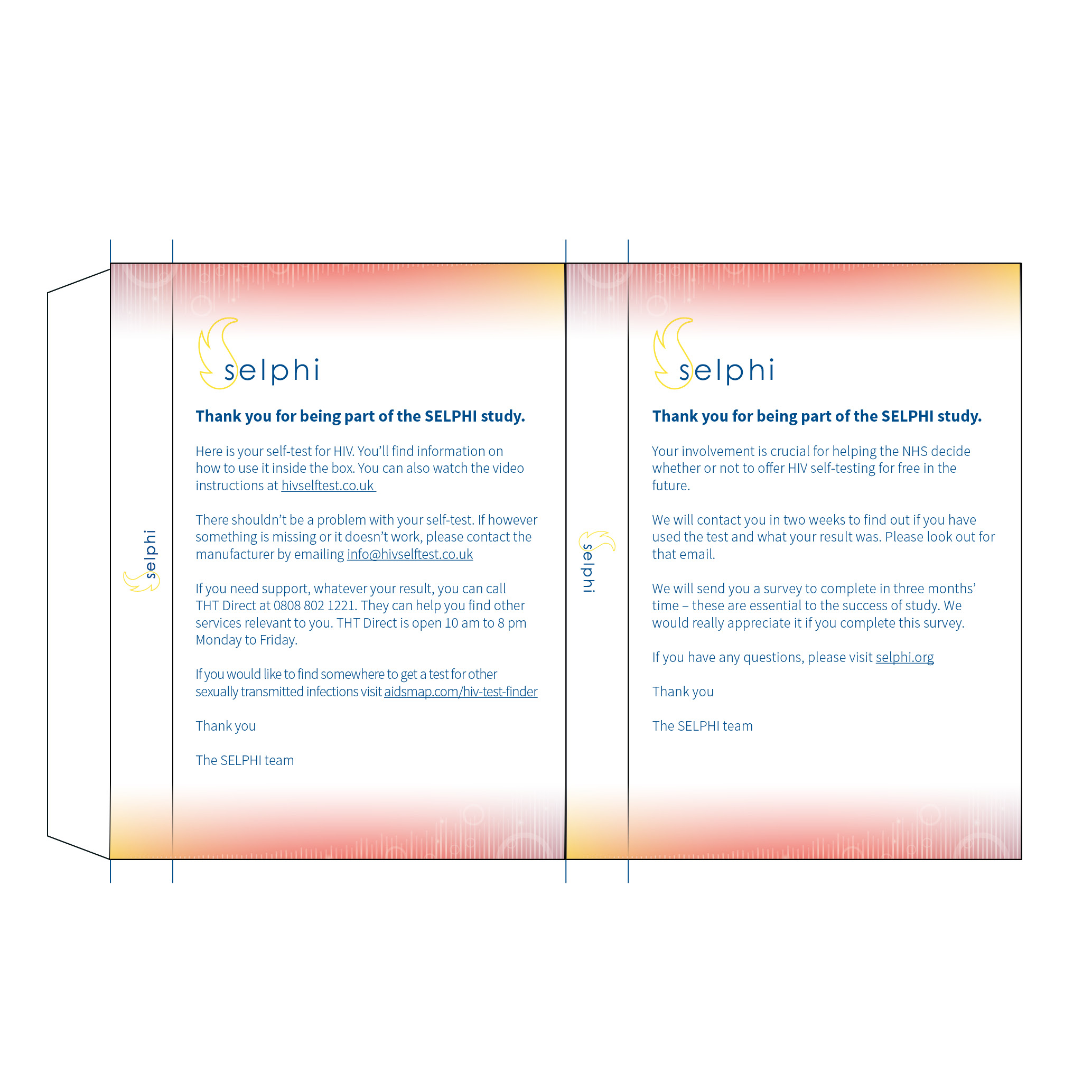

Supplement: Supplementary file 1 — Kit sleeve design. (JPG 519 kb) [file 12879_2019_4247_MOESM1_ESM.jpg]
